# Multimodal Quantitative MRI Reveals No Evidence for Tissue Pathology in Idiopathic Cervical Dystonia

**DOI:** 10.3389/fneur.2019.00914

**Published:** 2019-08-27

**Authors:** René-Maxime Gracien, Franca Petrov, Pavel Hok, Alexandra van Wijnen, Michelle Maiworm, Alexander Seiler, Ralf Deichmann, Simon Baudrexel

**Affiliations:** ^1^Department of Neurology, Goethe University, Frankfurt, Germany; ^2^Brain Imaging Center, Goethe University, Frankfurt, Germany; ^3^Department of Neurology, Palacký University Olomouc and University Hospital Olomouc, Olomouc, Czechia

**Keywords:** idiopathic dystonia, quantitative MRI, relaxometry, proton density, movement disorders

## Abstract

**Background:** While in symptomatic forms of dystonia cerebral pathology is by definition present, it is unclear so far whether disease is associated with microstructural cerebral changes in idiopathic dystonia. Previous quantitative MRI (qMRI) studies assessing cerebral tissue composition in idiopathic dystonia revealed conflicting results.

**Objective:** Using multimodal qMRI, the presented study aimed to investigate alterations in different cerebral microstructural compartments associated with idiopathic cervical dystonia *in vivo*.

**Methods:** Mapping of T_1_, T_2_, ${\text{T}}_{2}^{*}$, and proton density (PD) was performed in 17 patients with idiopathic cervical dystonia and 29 matched healthy control subjects. Statistical comparisons of the parametric maps between groups were conducted for various regions of interest (ROI), including major basal ganglia nuclei, the thalamus, white matter, and the cerebellum, and voxel-wise for the whole brain.

**Results:** Neither whole brain voxel-wise statistics nor ROI-based analyses revealed significant group differences for any qMRI parameter under investigation.

**Conclusions:** The negative findings of this qMRI study argue against the presence of overt microstructural tissue change in patients with idiopathic cervical dystonia. The results seem to support a common view that idiopathic cervical dystonia might primarily resemble a functional network disease.

## Introduction

Idiopathic focal dystonias are movement disorders of unknown cause defined by presence of sustained or intermittent muscle contractions causing abnormal, often repetitive movements, postures or both, which affect a single body region (1). The most frequent forms include cervical dystonia, blepharospasm, writer's cramp, spasmodic dysphonia, oromandibular dystonia, and Meige syndrome (1, 2). The clinical manifestations are similar to the acquired forms of focal dystonia, which may be caused by a single or multiple macroscopic lesions of vascular, traumatic, toxic, infectious, or neoplastic origin (1) in the putamen, caudate nucleus, globus pallidus (3), or posterolateral thalamus (4).

While in symptomatic forms of dystonia cerebral pathology is by definition present, it is not yet clear whether development of idiopathic dystonia is also driven by microstructural cerebral changes as most histopathological studies either found no overt pathology, or have not yet been replicated (2). None of the previous histopathological studies found any abnormality in the basal ganglia (5–9).

Although histopathological studies are crucial for understanding the cellular mechanism leading to manifestation of dystonia, they are commonly restricted to a relatively small sample size and/or analysis of a fraction of the cerebral tissue due to methodological constraints. Non-invasive imaging methods, especially magnetic resonance imaging (MRI), do not share this limitation. However, previous imaging studies in idiopathic dystonia have failed so far to provide a definitive answer as to the presence and localization of morphological abnormalities (10, 11). For instance, an increased gray matter (GM) volume has been observed in the striatum and cerebral cortex in some patient cohorts (12, 13), whereas other studies have reported widespread decreases of GM volume (14–16). Importantly, most previous studies are based on conventional MRI techniques showing mixed signal contrasts, which cannot be easily linked to the underlying microstructural tissue changes. Inconsistencies among the studies may arise from hardware-specific factors that affect conventional MRI techniques (10, 17). Well-defined physical parameters unaffected by hardware-specific bias, including T_1_, T_2_, and ${\text{T}}_{2}^{*}$ relaxation times, and proton density (PD), can be obtained using quantitative MRI (qMRI) (18). These qMRI parameters can be more directly attributed to certain microstructural tissue properties, which makes them promising candidates for investigations of patients with neurological disorders in general.

In fact, the majority of previous qMRI studies in idiopathic dystonia have employed diffusion tensor imaging (DTI) to probe for changes in tissue microstructure via the measurement of parameters that are related to the diffusion of water molecules in tissue (19–23). In these studies, diffusion changes have been reported for various brain regions such as the basal ganglia, cerebellum, motor cortex, and white matter tracts. However, findings are highly heterogeneous and also partly inconsistent across studies (24, 25).

So far, only two previous studies have used MRI relaxometry for the assessment of cerebral tissue composition in idiopathic focal dystonia, both examining patients with idiopathic cervical dystonia. The first study reported increased T_2_ relaxation times in the putamen and globus pallidus, which were attributed to focal cell loss and subsequent gliosis resulting in increased water content (26). In contrast, the more recent study has demonstrated decreased ${\text{T}}_{2}^{*}$ values in the globus pallidus, suggesting an increased iron content associated with the disease (27). T_2_ and ${\text{T}}_{2}^{*}$ likely reflect microstructural properties such as the iron and myelin content, which affect both T_2_ and ${\text{T}}_{2}^{*}$ relaxation times in a similar way (28–30). Thus, interpretation of the results of the previous qMRI studies showing T_2_ increases in one study and ${\text{T}}_{2}^{*}$ decreases in the other study in idiopathic focal dystonia is challenging (26, 27). To our knowledge, there have been no further studies employing MR-relaxometry in idiopathic dystonias. Therefore, it remains unknown so far whether other qMRI parameters, such as the T_1_ relaxation time or PD, are also affected.

To address these issues, we used a multimodal qMRI protocol employing T_1_, T_2_, ${\text{T}}_{2}^{*}$, and PD mapping in order to simultaneously assess multiple tissue characteristics that are potentially affected in patients with idiopathic cervical dystonia. The major goal of this study was to investigate whether one or several of the brain tissue relaxation times T_1_, T_2_, ${\text{T}}_{2}^{*}$, or the PD differ between patients with idiopathic cervical dystonia and healthy subjects. To this end, group comparisons of qMRI parameter maps were performed using techniques for whole brain voxel-wise statistical analysis and, additionally, using a region of interest (ROI)-based approach. With respect to the latter, averaged T_1_, T_2_, and ${\text{T}}_{2}^{*}$ relaxation times and PD were derived from the respective parameter maps for several subcortical and cerebellar ROIs, which are thought to be primarily involved in the pathophysiology of the disease (2, 10).

## Materials and Methods

### Participants

Twenty patients with idiopathic cervical dystonia were initially recruited for this study. Datasets from three patients were discarded due to movement artifacts, so the analysis comprised 17 patients (nine females). All patients received botulinum toxin A as part of their regular treatment. The degree of disability was rated using the Tsui Scale (31). Furthermore, 29 age- and gender-matched healthy subjects participated in the study (15 females). The approval by the institutional ethics committee (Ethik-Kommission des Fachbereichs Medizin der Goethe-Universität Frankfurt am Main, Germany) was obtained and all participants gave their written informed consent before taking part in the study.

The MRI acquisition was performed on a 3-Tesla whole body scanner (Magnetom TRIO MR scanner, Siemens Medical Solutions, Erlangen, Germany), equipped with an 8-channel phased-array head coil for signal reception and a body coil for radio frequency (RF) transmission.

The following measures were taken to reduce movement artifacts: Scans were conducted ~2 weeks after the last treatment with botulinum toxin when satisfactory treatment effects were already present in most patients. None of the examined patients suffered from severe head tremor. Furthermore, the head was comfortably bolstered in the coil to reduce movements.

### Data Acquisition

T_1_, T_2_, ${\text{T}}_{2}^{*}$, and PD maps are the results of specific procedures that measure actual tissue parameters for each single voxel (for example using exponential fitting for T_2_ mapping) and apply corrections for hardware effects such as transmit field inhomogeneities, the receiver bias, and B_0_ inhomogeneities. As opposed to conventional T_1_/T_2_(^*^)/PD-weighted images, the respective qMRI maps represent “pure” T_1_/T_2_(^*^)/PD contrasts. The value of each single voxel in the parameter maps represents a physical quantity that can be used for statistical testing.

T_1_ and PD mapping were based on the variable flip angle (VFA) method (32). The technique requires two spoiled gradient echo (GE) datasets acquired at different excitation angles α_1_ and α_2_, the smaller angle yielding stronger PD weighting, the larger angle stronger T_1_ weighting. Acquisition parameters were: scan duration: 9:48 min, TE/TR/α_1_/α_2_ = 6.7 ms/16.4 ms/4°/24°, bandwidth (BW) = 222 Hz/Pixel, field-of-view (FoV) = 256 × 224 × 160 mm^3^, resolution = 1 × 1 × 1 mm^3^. A special readout scheme was used to increase the signal-to-noise ratio (33), acquiring two gradient echoes with different degrees of phase encoding after each excitation pulse.

Mapping of non-uniformities of the transmitted radiofrequency (RF) field (B_1_) was performed as described previously (34). The method is based on the acquisition of two GE datasets, one of which is preceded by an RF pulse (nominal angle: 45°) which causes a B_1_-dependent reduction of the longitudinal magnetization and therefore of the signal intensity. The parameters were: scan duration: 0:53 min, TE/TR/α = 5 ms/11 ms/11°, BW = 260 Hz/Pixel. FoV as above, resolution: 4 × 4 × 4 mm^3^.

Furthermore, two GE datasets with different TE were recorded. These were required for correcting residual signal losses induced by ${\text{T}}_{2}^{*}$ relaxation. The parameters were: scan duration: 5 min, TE_1_/TE_2_/TR/α = 4.3 ms/11 ms/1,336 ms/50°, BW = 292 Hz/Pixel. FoV as above, resolution = 2 × 2 × 2 mm3.

For ${\text{T}}_{2}^{*}$ and B_0_ mapping, eight multiple-echo GE datasets with export of modulus and phase data were acquired: Scan duration: 5:46 min, TE_1−8_ = [10, 16, 22, 28, 34, 40, 46, 52] ms, TR/α = 2,400 ms/30°, BW = 299 Hz/Pixel, 40 slices, 2 mm slice thickness with 1 mm inter-slice gap, FoV: 240 × 180 mm^2^, resolution = 1.25 × 1.25 mm^2^. The sequence was repeated with 50% and 25% resolution to correct for motion artifacts as explained in the literature (35) (scan durations: 3:07 and 1:41 min).

T_2_ mapping was based on the acquisition of four fast spin echo datasets with different TE: scan duration: 8:08 min, TE = [17, 86, 103, 120] ms, TR = 8 s, BW = 100 Hz/Pixel, FoV: 240 × 180 mm^2^, matrix size: 192 × 144, 40 axial slices with a thickness of 2 mm, inter-slice gap of 1 mm, spatial resolution: 1.25 × 1.25 mm^2^, turbo factor: 11, refocusing angle: 180°.

### Data Analysis

Data analyses were implemented with custom-written Perl, Bash, and MATLAB scripts applying functions from FSL 5.0.7 (FMRIB, Oxford, UK) (36), FreeSurfer 6.0.1 (Athinoula A. Martinos Center for Biomedical Imaging, Boston, MA, USA) (37) and MATLAB (MathWorks, Natick, MA, USA).

#### Calculation of T_1_, T_2_, ${\text{T}}_{2}^{*}$, and PD Parameter Maps

The VFA method was used for mapping of T_1_ (32). Data were corrected for B_0_ and B_1_ inhomogeneities and for the effect of insufficient spoiling of transverse magnetization as described previously (38). Beforehand, B_0_ maps were calculated from the respective GE phase datasets that had been acquired at different TE using FSL PRELUDE and FUGUE. B_1_ was obtained according to a method described by Volz et al. (34).

Maps of T_2_ and ${\text{T}}_{2}^{*}$ relaxation times were calculated by voxel-wise exponential fitting of the TE dependence of signal levels in the respective datasets. Correction for movement artifacts was included for ${\text{T}}_{2}^{*}$ mapping as described by Nöth et al. (35). Furthermore, the ${\text{T}}_{2}^{*}$ maps were corrected for macroscopic B_0_ distortions according to a previous publication (39). The T_2_ maps were corrected for the effects of stimulated and secondary echoes occurring in the fast spin echo datasets, using the method described previously (40).

PD mapping was performed as described by Volz et al. (41). Subsequently, the PD weighted GE-data were corrected for B_1_ inhomogeneities and for T_1_ and ${\text{T}}_{2}^{*}$ effects. Furthermore, a correction for inhomogeneities of the receive coil sensitivity profile was performed via bias field correction (41).

For the purpose of subsequent normalization and tissue segmentation, synthetic magnetization-prepared rapid gradient-echo (MP-RAGE) datasets with mixed T_1_/PD-weighting were derived from the T_1_ maps as described in the literature (42, 43), calculating pseudo PD maps from the T_1_ values (44). The following acquisition parameters were assumed: TR = 1,900 ms, TI = 900 ms, FoV = 256 × 224 × 160 mm^3^, resolution: 1 × 1 × 1 mm3, α = 9°, echo spacing = 8.1 ms, 192 phase encoding steps inside the inner loop with symmetric *k*-space coverage.

#### Whole Brain Statistical Analysis

For whole brain voxel-wise statistical comparisons, data were normalized into Montreal Neurological Institute (MNI) 152 space according to the following steps: T_2_ and ${\text{T}}_{2}^{*}$ maps were first coregistered to the synthetic MP-RAGE data with FSL FLIRT. Please note, that T_1_ and PD maps are already in the same space as the synthetic anatomies. Synthetic MP-RAGE data were normalized into MNI-space using FSL FNIRT after initialization with FSL FLIRT. The resulting coregistration matrices were then used to (co-)normalize the qMRI parameter maps.

For each qMRI parameter map (T_1_, T_2_, ${\text{T}}_{2}^{*}$, and PD), voxel-wise statistical comparisons between groups were performed with FSL “randomize” using unpaired *t-*tests and threshold-free cluster enhancement for correction of multiple comparisons. Voxels with the value zero in any dataset were excluded from the analysis.

#### ROI-Based Statistics

For ROI-based statistics of qMRI parameters, tissue segmentation of the synthetic MP-RAGE datasets (43) was performed with the “recon-all” stream implemented in the FreeSurfer toolbox. Masks of the putamen, pallidum, thalamus, and caudate nucleus were extracted from the FreeSurfer results for the right and the left hemisphere. As head movements are represented bilaterally in the basal ganglia and in the motor cortex (45), masks from both hemispheres were combined. Furthermore, cerebellar WM/cortex masks with bihemispheric coverage were extracted. To avoid partial voluming from cerebrospinal fluid (CSF) compartments, voxels with T_1_ values above 2,000 ms were removed from all masks. For ROI-based T_2_ and ${\text{T}}_{2}^{*}$ analyses, the masks were further coregistered to the T_2_ and ${\text{T}}_{2}^{*}$ maps using inverted coregistration matrices from the previous registration between the T_2_/${\text{T}}_{2}^{*}$ maps and the synthetic MP-RAGE data. To reduce partial voluming effects related to this coregistration, voxels with T_2_ values above an empirically defined threshold of 200 ms and voxels with ${\text{T}}_{2}^{*}$ values exceeding 100 ms were removed from the respective masks for each participant.

Furthermore, a cerebral WM mask was created using the segmentation tool FSL FAST. All voxels overlapping either with subcortical or cerebellar masks were removed. To reduce partial voluming, the WM mask was further eroded with a 3 × 3 × 3 mm3 kernel.

Averaged T_1_, T_2_, ${\text{T}}_{2}^{*}$, and PD values were derived from the respective parameter maps for each bihemispheric ROI and statistical comparisons between groups were performed using non-parametric testing (Mann–Whitney–U, SPSS Statistics, Version 22.0.0). qMRI data from subcortical ROIs were further analyzed separately for each hemisphere. In addition, volumes of deep GM and cerebellar regions measured with FreeSurfer were also compared between groups.

## Results

The group of patients with cervical dystonia and the healthy control subjects were not different in terms of age (dystonia: 51.0 ± 8.9 years, control subjects: 50.5 ± 10.4 years; unpaired *t-*test: *p* = 0.86). The average Tsui score amounted to 5.2 ± 2.7 (range 1–10).

**Figure 1:**
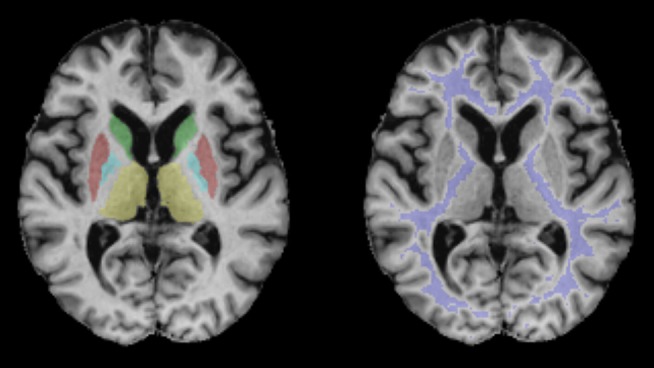
Regions of interest used for the evaluation of qMRI data demonstrated for a representative subject in MNI 152 standard space (*z* = 8). Blue: pallidum; red: putamen; green: caudate nucleus; violet: white matter; yellow: thalamus.

**Figure 2:**
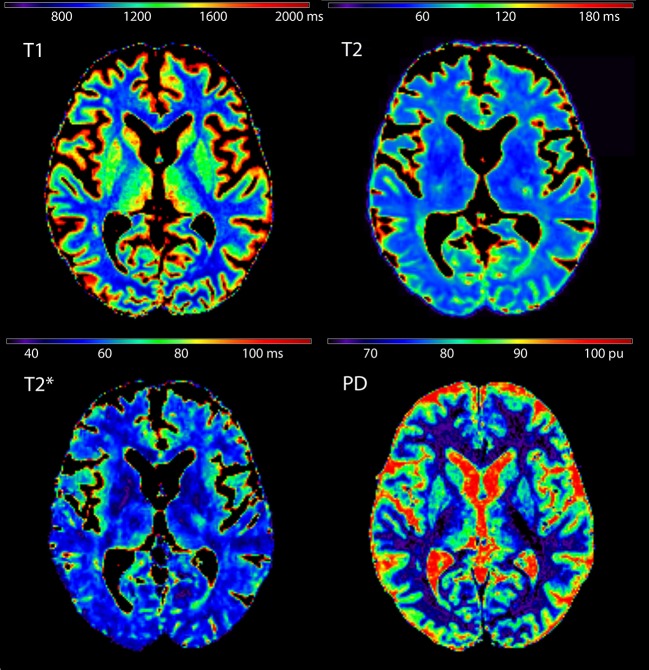
Quantitative T_1_, T_2_, ${\text{T}}_{2}^{*}$, and proton density maps shown for the same subject presented in Figure 1 (*z* = 8).

ROIs used for the extraction of qMRI values are presented in Figure 1 for a representative subject in MNI 152 standard space (*z* = 8). Figure 2 shows quantitative T_1_, T_2_, ${\text{T}}_{2}^{*}$, and PD maps of the same subject presented in Figure 1.

Whole brain voxel-wise analysis did not unveil any significant difference between patients with idiopathic cervical dystonia and healthy subjects for any qMRI parameter (T_1_, T_2_, ${\text{T}}_{2}^{*}$, or PD).

**Figure 3:**
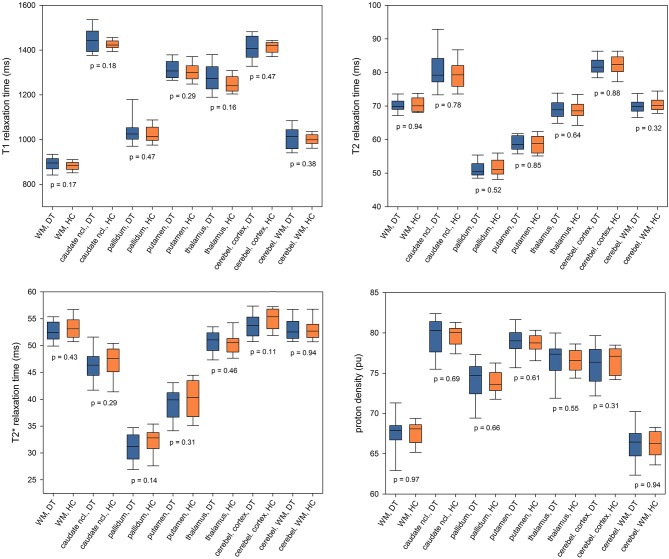
Region of interest-based analysis of T_1_, T_2_, and ${\text{T}}_{2}^{*}$ relaxation times and proton density. qMRI values were averaged across bilateral ROIs and presented as boxplots (median, upper, and lower quartile and 90% CI) for the patients with idiopathic cervical dystonia (DT) and healthy control subjects (HC). *P*-values for between-group comparisons are inserted into the diagram below the corresponding boxplots. Ncl., nucleus; cerebel., cerebellum.

qMRI results for the bilateral ROIs are demonstrated in Figure 3 as boxplots (median, upper and lower quartiles and 90% CI) for both groups. For none of the qMRI parameters any significant group difference was observed (T_1_: *p* ≥ 0.16, T_2_: *p* ≥ 0.32; ${\text{T}}_{2}^{*}$: *p* ≥ 0.11; PD: *p* ≥ 0.31). Evaluation of subcortical ROIs for each hemisphere separately also yielded only negative findings (Supplementary Table 1). There was no group difference with respect to deep GM or cerebellar volumes (Supplementary Table 2).

## Discussion

Using multimodal quantitative MRI, we did not observe significant group differences with respect to cerebral T_1_, T_2_, ${\text{T}}_{2}^{*}$ relaxation times, and PD between patients with idiopathic cervical dystonia and healthy control subjects.

Previous conventional MRI studies have demonstrated subtle differences in the volume in multiple brain structures in patients with idiopathic dystonia. However, there is little agreement among these studies (10): Increased (12, 13) and decreased GM volume in various brain regions (14–16) or even a combination of both (46–48) have been observed. The discrepancies between these imaging studies seem to suggest that volume changes in idiopathic dystonia are—if present at all—rather small and, thus, difficult to detect in small cohorts. However, some of the discrepancies may also be explained by the fact that conventional MRI techniques rely on mixed signal contrasts (10) and are thus affected by hardware-specific factors and artifacts.

In contrast to conventional MRI, qMRI techniques provide tissue parameters that are unaffected by hardware effects. Thus, in qMRI, a more direct link can be established between the imaging parameters, such as PD, T_1_, T_2_, and ${\text{T}}_{2}^{*}$, and the underlying microstructural changes. PD mainly reflects the tissue water content (49). The longitudinal relaxation time T_1_ also provides information about the water content (50, 51), but is additionally related to iron content (51), the degree of myelination (52), and the degree of gliosis and axonal damage (53). T_2_ is primarily considered to be a marker of myelin content, but is also affected by iron and water proportions (54). ${\text{T}}_{2}^{*}$ provides the most direct information about the tissue iron content (55).

Despite using quantitative parameters, our results differ from the findings in the two previous qMRI studies using T_2_ and ${\text{T}}_{2}^{*}$ mapping to investigate patients with cervical dystonia. While the study by Schneider et al. (26) reported a prolongation of T_2_ relaxation time in the putamen and globus pallidus, another study observed decreased ${\text{T}}_{2}^{*}$ values in the globus pallidus (27). Increased T_2_ relaxation times in the basal ganglia nuclei were speculated to originate from cell loss and gliosis and the ${\text{T}}_{2}^{*}$ decrease was interpreted as the result of increased iron deposition exceeding the natural iron increase associated with aging. Since the number of included patients was similar in our study (17 patients) as compared to the two previous studies [17 patients in (26) and 12 patients in (27)], several possible reasons why previous results could not be confirmed in this study, such as differences in patient characteristics, should be considered. However, subjects included in all three studies were of similar average age, i.e., 45.4 (27), 49.7 (26), and 51.0 years (present study). The average disease duration in our study was 11.2 years, while it was 8.2 (27) and 6.4 years (26) in the previous studies. In summary, the study populations were similar enough not to consider the demographic differences as a significant factor that would explain different study conclusions.

Several methodological differences can be identified that might account for the inconsistent results. For instance, the contribution of partial volume effects may vary across the studies. This could especially affect studies utilizing acquisition techniques with relatively lower spatial resolution, e.g., the study by Schneider et al. (26) evaluating images with 5-mm slice thickness and 7.5-mm gaps between slices. Further systematic differences may arise from the method for ROI selection. The ROIs were chosen manually in one previous study (26), while an automated segmentation approach was used by Aschermann et al. (27) and in our study. However, even automated segmentation tools, such as FIRST in FSL (56) or FreeSurfer (57), may yield different results as they employ different algorithms and independent training datasets. Being aware of the limitations of segmentation methods, efforts were taken in the present study to minimize the partial volume effects as described in the Materials and Methods section. Taken together, using multimodal qMRI techniques with a high spatial resolution and taking efforts to reduce partial volume effects, no changes in qMRI parameters could be observed in the present study.

The lack of significant group differences in qMRI parameters observed in this study together with the heterogeneous findings in previous conventional (10), DTI (19–23), and relaxometry studies (26, 27) as well as the high variability of histological findings (2, 5–9) raises the question of whether idiopathic focal dystonias share a common site of microscopic pathology at all. As a matter of fact, it has been previously suggested that idiopathic focal dystonias may be considered as a purely functional disorder (10, 11, 24, 58, 59). Network-wide differences in brain activation that disappear with successful symptomatic treatment have been observed in functional MRI (60–62).

A previous histopathological study observed a reduced density of Purkinje cells in cervical dystonia (9) suggesting that the cerebellum might be a candidate region exhibiting common histopathological changes, while the applied qMRI techniques did not unveil cerebellar changes in the presented investigation. Future combined histological and MRI studies might help to explain this discrepancy and investigate the relationship between histological findings and qMRI parameters more closely.

A limitation of this study is the relatively small sample size, especially in the context of a negative result. However, 17 patients with focal cervical dystonia have been included in the analysis and this number lies in the range of previous qMRI studies in this field. According to a standard power calculation, a sample size of 17 is sufficient to detect an effect of the size of the sample standard deviation with a power of 0.83 (assuming a two-tailed *t-*test). This renders the presence of a larger parameter change rather unlikely. In this context, it is also worth to consider inherent accuracy limits of the method, which *per se* hamper the detection of smaller parameter changes (i.e., those lying below the standard deviation). For example, a scan-rescan variability of ~3% was observed for T_1_ measurements in Nöth et al. (42), and an even higher variability can be assumed for T_2_ and ${\text{T}}_{2}^{*}$ measurements (63, 64). Nevertheless, future studies would surely benefit from larger sample sizes and a correlation of MRI with histologic data in order to clarify some of the inconsistencies across the existing studies.

In conclusion, assessment of patients with idiopathic cervical dystonia with modern multimodal qMRI and segmentation techniques did not unveil any changes in tissue composition. The results seem to support the view that idiopathic cervical dystonia might be primarily a functional network disease, albeit the existence of tissue changes that lie below the accuracy of the method cannot be ruled out at the moment.

## Data Availability

The raw data supporting the conclusions of this manuscript will be made available by the authors, without undue reservation, to any qualified researcher upon reasonable request.

## Ethics Statement

This study was carried out in accordance with the recommendations of the local ethics committee (Ethik-Kommission des Fachbereichs Medizin der Goethe-Universität Frankfurt am Main, Germany) with written informed consent from all subjects. All subjects gave written informed consent in accordance with the Declaration of Helsinki. The protocol was approved by the local ethics committee.

## Author Contributions

R-MG, PH, AS, RD, and SB contributed to the conception and design of the study. R-MG, FP, RD, and SB organized the study. R-MG, FP, AvW, and MM executed the study and acquired the data. R-MG and SB designed the statistical analysis. R-MG and FP performed the statistical analysis. R-MG, PH, AvW, MM, AS, and SB reviewed the statistical analysis. RD derived the parameter maps from the source data. R-MG, FP, PH, and SB wrote the first draft and sections of the manuscript. All authors contributed to manuscript revision, read, and approved the submitted version.

### Conflict of Interest Statement

RD received compensation as a Consultant for MR scanner procurement by the Wellcome Trust Centre for Neuroimaging, UCL, London, UK. The remaining authors declare that the research was conducted in the absence of any commercial or financial relationships that could be construed as a potential conflict of interest.
