# Supplementary material for: Multimodal Quantitative MRI Reveals No Evidence for Tissue Pathology in Idiopathic Cervical Dystonia
Source: Front Neurol. 2019 Aug 27;10:914. doi: 10.3389/fneur.2019.00914 (PMC6719627; doi:10.3389/fneur.2019.00914)
Supplement: Supplementary file 1 [file Data_Sheet_1.docx]

Supplementary Material

# Supplementary Table 1

**Supplementary Table 1.** Group comparison of quantitative magnetic resonance imaging (qMRI) parameters. For each region of interest, mean values and standard deviations for both groups are provided, as well as the p value of Mann-Whitney U test. Abbreviations: LH – left hemisphere; PD – proton density; RH – right hemisphere; T_1_ – T_1_ relaxation time; T_2_ – T_2_ relaxation time; T_2_* – T_2_* relaxation time; WM – white matter.

| Parameter | Region of interest | Patients *n* = 17 | Control subjects *n* = 29 | *p*-value |
| --- | --- | --- | --- | --- |
| T_1_ [ms] | Caudate nucleus, LH | 1445.88 (57.96) | 1423.56 (34.03) | 0.37 |
|  | Caudate nucleus, RH | 1446.31 (64.53) | 1423.82 (35.21) | 0.14 |
|  | Globus pallidus, LH | 1035.78 (63.69) | 1015.58 (52.68) | 0.19 |
|  | Globus pallidus, RH | 1042.35 (70.00) | 1031.01 (44.05) | 0.88 |
|  | Putamen, LH | 1304.59 (54.12) | 1289.77 (53.79) | 0.32 |
|  | Putamen, RH | 1327.67 (56.34) | 1314.36 (41.85) | 0.53 |
|  | Thalamus, LH | 1261.27 (64.06) | 1231.54 (41.14) | 0.11 |
|  | Thalamus, RH | 1296.21 (67.72) | 1270.05 (40.37) | 0.29 |
| T_2_ [ms] | Caudate nucleus, LH | 80.48 (6.56) | 78.48 (4.41) | 0.33 |
|  | Caudate nucleus, RH | 80.72 (6.82) | 80.24 (5.25) | 0.81 |
|  | Globus pallidus, LH | 51.41 (2.73) | 52.26 (3.22) | 0.37 |
|  | Globus pallidus, RH | 51.01 (2.13) | 50.90 (2.74) | 0.90 |
|  | Putamen, LH | 59.36 (2.93) | 59.28 (3.86) | 0.96 |
|  | Putamen, RH | 58.43 (2.24) | 58.40 (3.15) | 0.83 |
|  | Thalamus, LH | 68.95 (3.04) | 68.33 (2.90) | 0.59 |
|  | Thalamus, RH | 69.08 (3.00) | 69.08 (3.32) | 0.90 |
| T_2_* [ms] | Caudate nucleus, LH | 46.34 (3.83) | 47.48 (3.57) | 0.13 |
|  | Caudate nucleus, RH | 46.38 (3.28) | 46.85 (4.24) | 0.66 |
|  | Globus pallidus, LH | 30.94 (2.85) | 32.33 (2.83) | 0.14 |
|  | Globus pallidus, RH | 30.81 (3.51) | 32.15 (3.08) | 0.14 |
|  | Putamen, LH | 39.18 (3.55) | 40.11 (4.13) | 0.41 |
|  | Putamen, RH | 38.88 (2.68) | 39.96 (3.57) | 0.31 |
|  | Thalamus, LH | 50.86 (2.72) | 50.65 (2.49) | 0.85 |
|  | Thalamus, RH | 50.54 (2.17) | 50.16 (2.47) | 0.63 |
| PD [pu] | Caudate nucleus, LH | 78.39 (2.73) | 78.76 (1.62) | 0.78 |
|  | Caudate nucleus, RH | 80.79 (2.51) | 80.53 (1.48) | 0.41 |
|  | Globus pallidus, LH | 73.79 (2.88) | 73.75 (1.80) | 0.79 |
|  | Globus pallidus, RH | 73.98 (3.22) | 74.04 (1.61) | 0.31 |
|  | Putamen, LH | 79.09 (2.10) | 78.79 (1.58) | 0.61 |
|  | Putamen, RH | 78.92 (2.42) | 78.78 (1.24) | 0.42 |
|  | Thalamus, LH | 76.47 (2.49) | 76.46 (1.75) | 0.79 |
|  | Thalamus, RH | 76.81 (2.75) | 76.60 (1.71) | 0.56 |

# Supplementary Table 2

**Supplementary Table 2.** Group comparison of volumes of regions of interest [mm^3^]. For each region of interest, mean values and standard deviations for both groups are provided, as well as the p value of Mann-Whitney U test. Abbreviations: LH – left hemisphere; RH – right hemisphere; WM – white matter.

| Region of interest | Patients *n* = 17 | Control subjects *n* = 29 | *p*-value |
| --- | --- | --- | --- |
| Caudate nucleus, LH | 3,362 (471) | 3,540 (409) | 0.30 |
| Caudate nucleus, RH | 3,546 (471) | 3,666 (482) | 0.72 |
| Globus pallidus, LH | 1,698 (269) | 1,799 (223) | 0.21 |
| Globus pallidus, RH | 1,665 (220) | 1,744 (246) | 0.42 |
| Putamen, LH | 4,630 (621) | 4,753 (566) | 0.86 |
| Putamen, RH | 4,696 (660) | 4,705 (655) | 0.86 |
| Thalamus, LH | 6,940 (805) | 7,090 (762) | 0.49 |
| Thalamus, RH | 6,776 (758) | 6,871 (792) | 0.99 |
| Cerebellar cortex | 104,450 (8,861) | 106,972 (13,894) | 0.72 |
| Cerebellar WM | 24,180 (3,175) | 25,654 (2,655) | 0.25 |
